# The 14-Kilodalton Human Growth Hormone Fragment a Potent Inhibitor of Angiogenesis and Tumor Metastasis

**DOI:** 10.3390/ijms24108877

**Published:** 2023-05-17

**Authors:** Baraah Tariq Shaker, Asmaa Anwar Ismail, Rawan Salih, Hassen Hadj Kacem, Mohamed Rahmani, Ingrid Struman, Khalid Bajou

**Affiliations:** 1 Department of Applied Biology, College of Sciences, University of Sharjah, Sharjah 27272, United Arab Emirates; u17105703@sharjah.ac.ae (B.T.S.); asmaa.ismail@sharjah.ac.ae (A.A.I.); u20104300@sharjah.ac.ae (R.S.); hkacem@sharjah.ac.ae (H.H.K.); 2 Human Genetics & Stem Cells Research Group, Research Institute of Sciences & Engineering, University of Sharjah, Sharjah 27272, United Arab Emirates; 3 Department of Molecular Biology and Genetics, College of Medicine & Health Sciences, Khalifa University, Abu Dhabi P.O. Box 127788, United Arab Emirates; mohamed.rahmani@ku.ac.ae; 4 Center for Biotechnology, Khalifa University, Abu Dhabi P.O. Box 127788, United Arab Emirates; 5 Laboratory of Molecular Angiogenesis, GIGA Research Center, University of Liège, 4000 Liège, Belgium; i.struman@uliege.be

**Keywords:** growth hormone, 14-kilodalton growth hormone fragment, tumor growth, metastasis, angiogenesis, plasminogen activator inhibitor-1

## Abstract

The 14-kilodalton human growth hormone (14 kDa hGH) N-terminal fragment derived from the proteolytic cleavage of its full-length counterpart has been shown to sustain antiangiogenic potentials. This study investigated the antitumoral and antimetastatic effects of 14 kDa hGH on B16-F10 murine melanoma cells. B16-F10 murine melanoma cells transfected with 14 kDa hGH expression vectors showed a significant reduction in cellular proliferation and migration associated with an increase in cell apoptosis in vitro. In vivo, 14 kDa hGH mitigated tumor growth and metastasis of B16-F10 cells and was associated with a significant reduction in tumor angiogenesis. Similarly, 14 kDa hGH expression reduced human brain microvascular endothelial (HBME) cell proliferation, migration, and tube formation abilities and triggered apoptosis in vitro. The antiangiogenic effects of 14 kDa hGH on HBME cells were abolished when we stably downregulated plasminogen activator inhibitor-1 (PAI-1) expression in vitro. In this study, we showed the potential anticancer role of 14 kDa hGH, its ability to inhibit primary tumor growth and metastasis establishment, and the possible involvement of PAI-1 in promoting its antiangiogenic effects. Therefore, these results suggest that the 14 kDa hGH fragment can be used as a therapeutic molecule to inhibit angiogenesis and cancer progression.

## 1. Introduction

Metastasis is the leading cause of increased mortality rates in cancer patients, accounting for 90% of cancer-related deaths [1]. During this complex multistep invasion-metastasis cascade, cancer cells invade distant organs through the lymphatic system or blood circulation by intravasation into blood vessels and then attachment to endothelial cells (ECs) that line blood vessels, allowing cancer cells to extravasate at secondary metastatic sites and penetrate tissues or organs, and then start to proliferate [2]. Thus, this process cannot be accomplished without neovascularization. In 1971, Judah Folkman discovered that tumor growth is angiogenesis-dependent and tumors cannot grow more than 1–2 mm^3^ in diameter without vascularization [3]. Angiogenesis plays a pivotal role in tumor metastasis development, as it aids in the formation of new blood vessels that supply tumor cells with oxygen and nutrients to sustain their growth and dissemination [4]. In addition, angiogenesis is associated with normal physiological processes vital for wound healing, embryogenesis, inflammation, and immune responses and contributes to pathological processes, including diabetes, inflammatory disorders, and cancer [5,6]. Angiogenesis occurs through distinct biological steps, including endothelial cell proliferation, migration, and organization into vessels. This cascade of events is tightly regulated by maintaining a balance between endothelial cells’ stimulatory pro- and antiangiogenic factors. This balance is disturbed by the prevalence of angiogenic factors in cancer, such as the vascular endothelial growth factor (VEGF), basic fibroblast growth factor (bFGF), and chemokines, which led to the concept of the ‘angiogenic switch’ [7,8,9]. Therefore, targeting angiogenesis in cancer therapy has become an attractive but challenging approach that will require the design and development of antiangiogenic molecules. 

Several studies have identified multiple angiogenic inhibitors that aim to prevent the growth of blood vessels; one of the angiogenic inhibitors that has been recently studied is the 16-kilodalton fragment of prolactin (16 kDa PRL) [10,11,12]. Prolactin belongs to the family of polypeptide hormones that also comprise growth hormone and placental lactogen. Members of this family share many similarities in structural and biological features [13]; thus, we are interested in testing a similar fragment from the growth hormone that has not been extensively studied. The 14-kilodalton human growth hormone (14 kDa hGH) N-terminal fragment is cleaved from its 22 kD full-length proangiogenic counterpart by thrombin and plasmin [14,15]. A high level of hGH was associated with an increase in cell proliferation and cancer promotion [16]. Targeting the hGH receptor reduces tumor progression and epithelial-mesenchymal transition in human melanoma cells [17]. However, the effect of 14k hGH-derived fragments on tumor melanoma or other types of cancers has not been investigated until now. This molecule belongs to the family of inhibins and attains antiangiogenic properties, unlike its native form; however, the role of this fragment in cancer remains not fully investigated [18,19]. In this study, we showed that 14 kDa hGH is capable of inhibiting tumor growth, angiogenesis, and metastasis and reducing angiogenesis in endothelial cells; we also showed that the antiangiogenic function of 14 kDa hGH is mediated by the plasminogen activator inhibitor-1 (PAI-1) molecule. Our data demonstrated, for the first time, the in vivo antitumoral, antiangiogenic, and antimetastatic effects of 14 kDa hGH. It was also suggested that the 14 kDa hGH fragment could be used as an antiangiogenic molecule to reduce primary cancer and block metastasis formation.

## 2. Results

### 2.1. The 14 kDa hGH Expression in Murine Melanoma and Endothelial Cell Lines 

**Figure 1:**
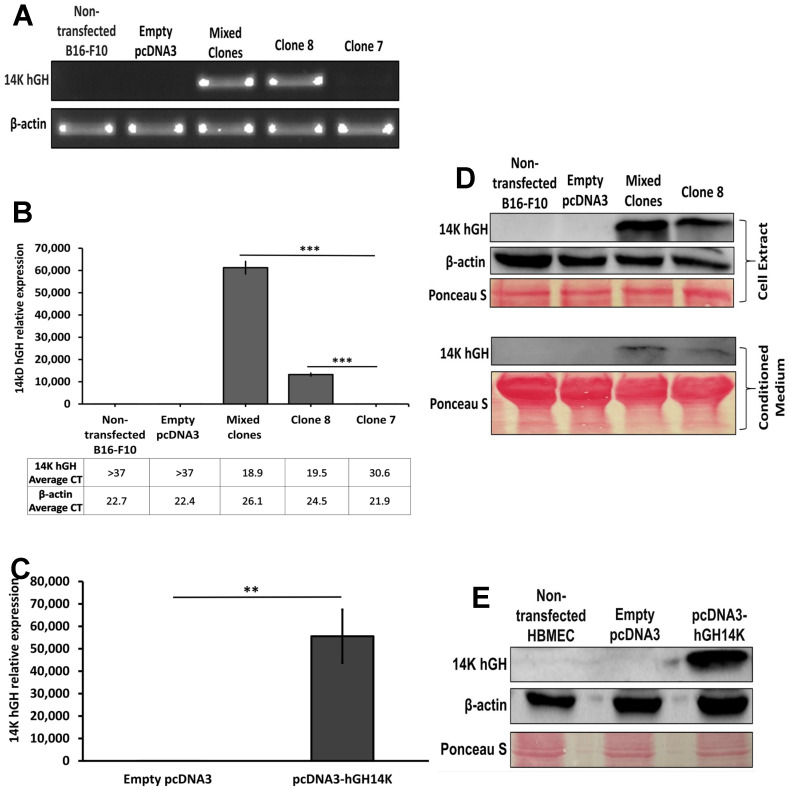
(**A**) Reverse transcription PCR of RNA extracted from B16-F10 murine melanoma cells transfected with pcDNA3-14 kDa hGH (mixed clones, clone 8, and clone 7), transfected with an empty plasmid (empty pcDNA3), and nontransfected cells (nontransfected B16-F10). Quantitative real-time PCR analysis of 14 kDa hGH gene expression level in stably transfected B16-F10 cells (mixed clones and clone 8) relative to clone 7. Ct average values of the 14 kDa hGH gene and beta-actin housekeeping gene for all clones are displayed below the *X*-axis (**B**) and transiently transfected HBME cells (**C**). Western blotting showed the expression of 14 kDa hGH protein in B16-F10 cell extract and conditioned medium (**D**) and HBME cell extract (**E**) following transfection with 14 kDa hGH or empty pcDNA3 vector. β-actin was used as the internal control, and Ponceau-S staining was used as the loading control. Statistically significant differences were calculated at ** *p* < 0.01 and *** *p* < 0.001.

To validate the expression of the 14 kDa hGH gene and protein in B16-F10 and HBME cells transfected with the 14 kDa hGH expression vector (pcDNA3-14 kDa hGH) compared to their corresponding cells transfected with the expression vector alone (pcDNA3), reverse transcription PCR and western blotting were carried out. Similarly, real-time quantitative PCR was performed to accurately quantify the expression level of 14 kDa hGH in both cell lines. Three B16-F10 stable clones (mixed clone, clone 8, and clone 7) showed clear bands corresponding to the 14 kDa hGH transcript, compared to controls (cells transfected with the empty pcDNA3 vector and nontransfected cells) (Figure 1A). The clones expressed varying levels of 14 kDa hGH mRNA, as shown in Figure 1B; two of the three clones, mixed clones, and clone 8, exhibited significantly higher expression of 14 kDa hGH in B16-F10 transfected cells. The average fold expression of the 14 kDa hGH gene in mixed clones and clone 8 was a 96- and 21-fold increase, respectively, relative to clone 7. The HBME cell lines showed a considerably high expression of the 14 kDa hGH gene following transient transfection with the 14 kDa hGH expression vector (Figure 1C). Western blot analyses confirmed the 14 kDa hGH expression at the protein level in B16-F10 clones and HBME cell lines, in concordance with the RT-PCR and RT-qPCR results. Cell extract and conditioned media from B16-F10 mixed clones and clone 8 demonstrated the presence of 14 kDa hGH proteins and showed particularly higher protein expression in mixed clones (Figure 1D). Furthermore, 14 kDa hGH protein was highly expressed in transiently transfected HBME cells with the 14 kDa hGH expression vector compared to the control, as shown in cell extract proteins (Figure 1E).

### 2.2. The 14 kDa hGH Reduces Cellular Proliferation and Colony-Forming Ability 

**Figure 2:**
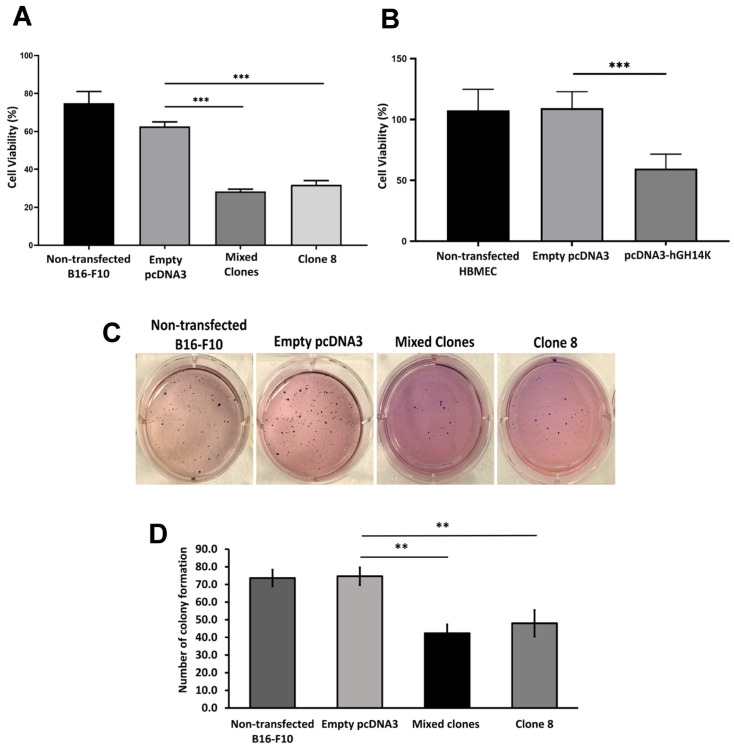
The effect of 14 kDa hGH on cell proliferation was evaluated by MTT assay at 48 h in B16-F10 cells (**A**) and HBME cells (**B**) after transfection with 14 kDa hGH or empty pcDNA3. (**C**) Representative colony formation assay after 21 days. (**D**) Quantitative analysis of the colony-forming ability of B16-F10 cells following transfection with 14 kDa hGH or empty pcDNA3. Data represent the mean ± SD of triplicate wells. Statistically significant differences were calculated at ** *p* < 0.01 and *** *p* < 0.001 versus empty pcDNA3 control.

In order to determine the effect of 14 kDa hGH on B16-F10 melanoma and HBME cell proliferation, cell viability was assessed using an MTT assay. After 48 h of incubation, the results revealed a significant decrease in cell viability of B16-F10 mixed clones and clone 8 cells compared to the controls (Figure 2A). Similarly, cell proliferation of transiently transfected HBME cells with the pcDNA3-14 kDa hGH plasmid was significantly reduced by approximately 50% compared to nontransfected cells or cells transfected with an empty pcDNA3 plasmid (Figure 2B), suggesting that 14 kDa hGH suppresses cell proliferation in cancer and endothelial cells. Moreover, the effect of 14 kDa hGH on tumorigenic inhibition of B16-F10 melanoma cells was assessed. Clonogenic assay results, interestingly, showed notable differences in the number of colony-forming units in B16-F10 cells, as illustrated in Figure 2C. The quantitative histogram results demonstrated that 14 kDa hGH significantly reduced the number of colony-forming units in B16-F10 mixed clones and clone 8 cells compared to the control cells (Figure 2D), elucidating the tumor suppressor function of 14 kDa hGH. Thus, colony formation assay results were consistent with the MTT assay results and further demonstrated that 14 kDa hGH remarkably reduces colony formation and cell viability.

### 2.3. The 14 kDa hGH Induces Cellular Apoptosis 

**Figure 3:**
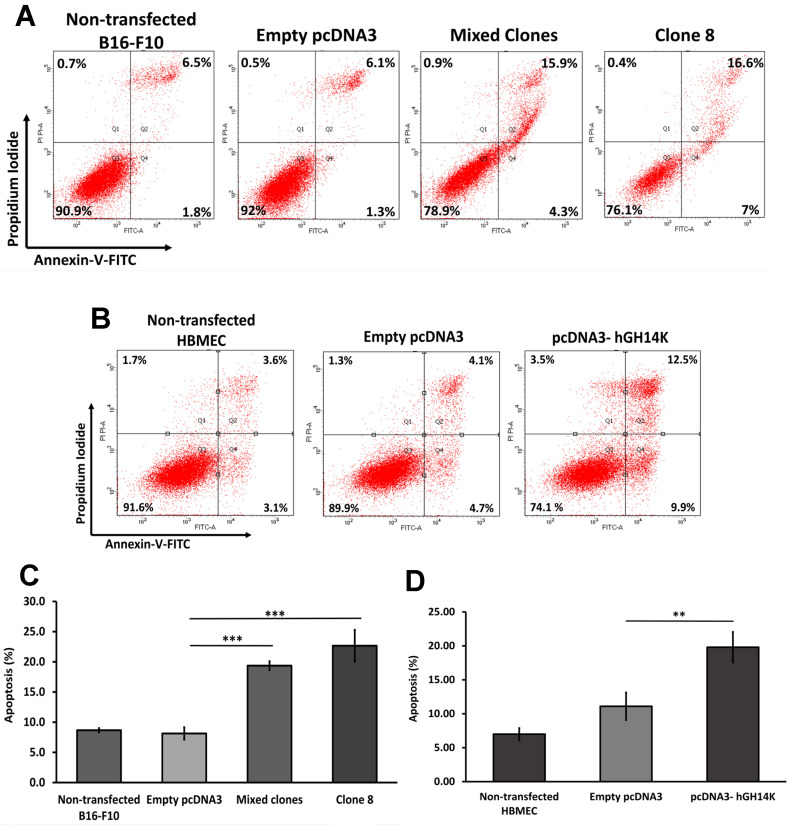
Detection of apoptosis induced by 14 kDa hGH using Annexin V-FITC/PI staining and FACS analysis. Representative scatter plots of B16-F10 cells (**A**) and HBME cells (**B**) after 48 h of transfection. The total percentage of apoptotic cells in each group of B16-F10 cells (**C**) and HBME cells (**D**) following transfection with 14 kDa hGH expression vector or the empty pcDNA3 vector. Data represent the mean ± SD of triplicate wells. Statistically significant differences were calculated at ** *p* < 0.01 and *** *p* < 0.001 versus empty pcDNA3 control. FITC, fluorescein isothiocyanate; PI, propidium iodide; FACS, fluorescence-activated cell sorting.

To investigate whether 14 kDa hGH mitigates cellular proliferation through the induction of cellular apoptosis, flow cytometry analysis was performed to measure the rate of apoptotic B16-F10 and HBME cells using Annexin V/PI staining. The results shown in Figure 3A,C revealed a statistically significant increase in early (Annexin V+) and late apoptosis (Annexin V+, PI+) of B16-F10 mixed clone and clone 8 cells, by 19% and 23%, respectively, compared to control cells that showed only 8% of apoptotic cells. Additionally, HBME cells transfected with pcDNA3 containing 14 kDa hGH showed a significant increase to 20% of apoptotic cells compared to 10% in control cells (Figure 3B,D), confirming that 14 kDa hGH induces apoptosis of endothelial and melanoma cells in vitro.

### 2.4. The 14 kDa hGH Mitigates Cellular Migration and Reduces Angiogenesis In Vitro 

**Figure 4:**
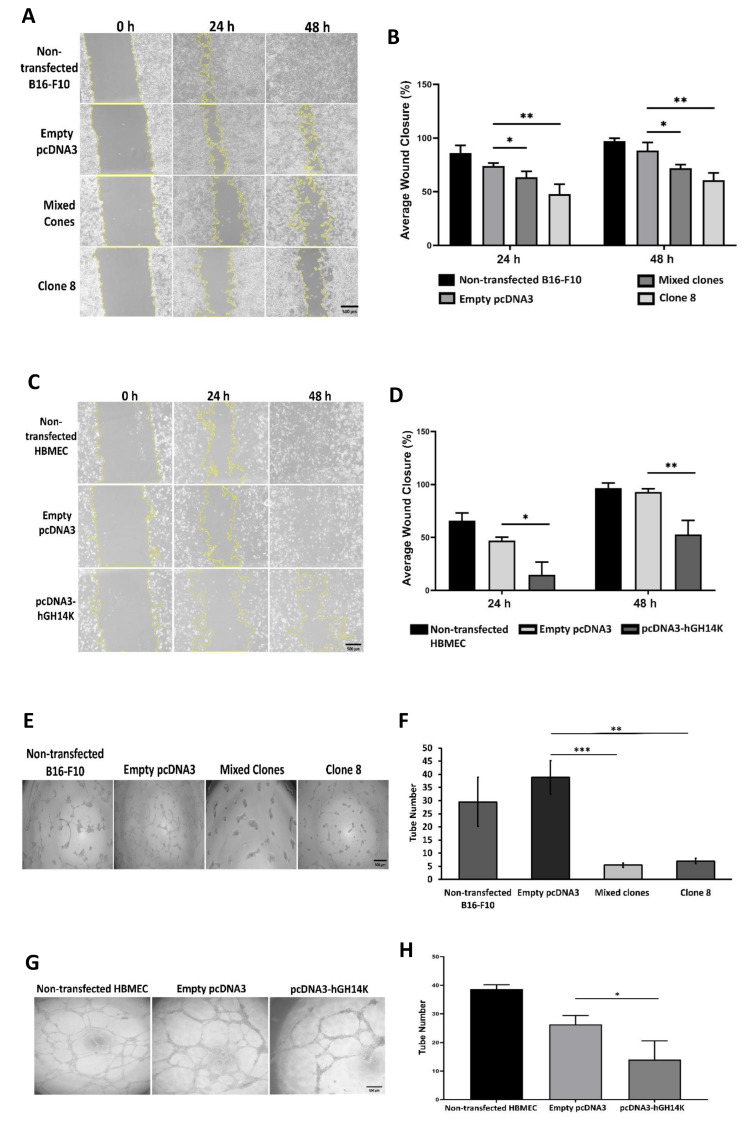
Representative pictures of the scratch assay using B16-F10 cells (**A**) and HBME cells (**C**) after 14 kDa hGH plasmid delivery in each group at 0 h, 24 h, and 48 h. Quantification of wound closure of B16-F10 cells (**B**) and HBME cells (**D**) transfected with 14 kDa hGH or empty pcDNA3. The histograms represent the percentage of wound closure at 24 h and 48 h relative to 0 h. Matrigel tube formation assay of stably transfected B16-F10 cells (**E**) and transiently transfected HBME cells (**G**) with 14 kDa hGH. The average number of branching formed by B16-F10 cells (**F**) and HBME cells (**H**) were determined and represented as histograms. Data represent the mean ± SD of triplicate wells. Statistically significant differences were calculated at * *p* < 0.05, ** *p* < 0.01, and *** *p* < 0.001 versus empty pcDNA3 control. (**A**,**C**,**E**,**G**) Scar bar = 500 µm.

Cellular migration is mandatory for cancer metastasis and essential for endothelial cells to form blood vessels during angiogenesis. An in vitro scratch assay was used to assess the effect of 14 kDa hGH on B16-F10 and HBME cells’ migration. Additionally, tumor cell vasculogenic activity in vitro was evaluated using a tube formation assay. Representative images in Figure 4A show that B16-F10 cells transfected with 14 kDa hGH migrated slower when compared to both non-transfected cells and ones transfected with the control empty pcDNA3 plasmid. In addition, the data showed a significant reduction of wound size after 48 h in B16-F10 mixed clones and clone 8 compared to the controls (Figure 4B). Likewise, fewer HBME cells transfected with 14 kDa hGH migrated compared to the control cells, with a significant deceleration in wound closure after 48 h of transfection (Figure 4C,D). Compared to the controls, B16-F10 mixed clones and clone 8 exhibited a notable inhibition of tube formation in the Matrigel-inducing tube formation assay, as illustrated in Figure 4E, and the data revealed a significant reduction in the number of branches in the network formed by mixed clones and clone 8 compared to the control cells (Figure 4F). As shown in Figure 4G,H, there was a significant disruption with a remarkable change in the morphology of tubes formed by HBME cells transfected with 14 kDa hGH compared to the control cells. Consistent with the effect of 14 kDa hGH on cell migration, these data demonstrate that 14 kDa hGH can reduce tube formation in vitro and suggest a potential antiangiogenic effect of 14 kDa hGH.

### 2.5. The 14 kDa hGH Inhibits Tumor Growth and Vascularization In Vivo 

**Figure 5:**
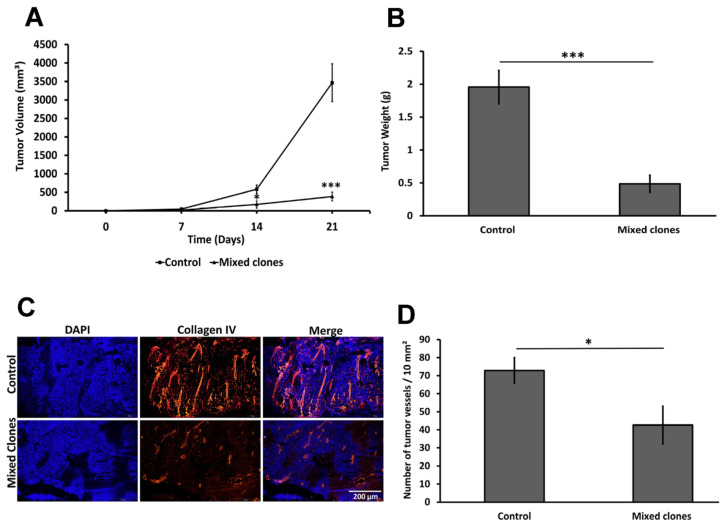
Tumors retrieved from C57BL/6 mice implanted with B16-F10 cells stably transfected with empty pcDNA3 vector (control) or 14 kDa hGH (mixed clones); tumors were monitored for 3 weeks, collected, and weighed. (**A**) Tumor growth kinetics, each line represents the average tumor growth over time for the control and mixed clone groups. (**B**) Average weight of B16-F10 tumors expressing stable 14 kDa hGH (n = 6) and control tumors with empty pcDNA3 plasmid (n = 7) 21 days after tumor implantation. (**C**) Representative histopathologic images of tumor tissues immunostained with collagen IV (red) and cell nuclei using DAPI (blue). Scale bars, 200 μm. (**D**) Quantification of tumor vessel number per 10 mm^2^ in tumor slides performed on tumor masses collected 3 weeks after tumor inoculation. Average tumor volume was calculated using the formula: length × width^2^ × 0.5, as mentioned in the material and methods section for the vessel quantification. Data represent mean ± SEM of eight to ten fields in each of the six mice. Statistically significant differences were calculated at * *p* < 0.05 and *** *p* < 0.001 versus control tumors with no 14 kDa hGH expression (empty pcDNA3 plasmid).

Considering the inhibitory effects of 14 kDa hGH on tumor cell proliferation, migration, and its proapoptotic function in vitro, we hypothesized that 14 kDa hGH might also reduce tumor development in vivo. Therefore, C57BL/6 mice were injected subcutaneously with either B16-F10 cells (1 × 10^6^) transfected with 14 kDa hGH or an empty pcDNA3 vector. Tumor growth was then monitored every week up to 21 days postinoculation in the two groups of mice. During the three-week follow-up period, a notable delay in tumor growth was observed in mice bearing the 14 kDa hGH-expressing tumors (n = 7) compared to control mice injected with control cells (n = 7). There is a significant reduction in tumor volume of 71% on Day 14 and 89% on Day 21 (Figure 5A). Mice were then sacrificed, and their tumors were harvested and weighed; the average tumor weight was significantly lower by 75% in the tumors expressing 14 kDa hGH compared to the control tumors (Figure 5B). Since we previously showed that 14 kDa hGH reduces in vitro angiogenesis, we investigated the effect of 14 kDa hGH on tumor angiogenesis. Tumor vascularization was evaluated in tumors expressing 14 kDa hGH and an empty pcDNA3 control. Collagen IV immunostaining, specific to the basement membrane of vessels, performed on tumor tissues, showed that the tumor blood vessels of 14 kDa hGH-bearing mice appeared smaller and collapsed compared to control mice (Figure 5C). In addition, the average number of tumor blood vessels determined in 10 mm^2^ was significantly reduced by 42% in 14 kDa hGH-expressing tumors compared to the control tumors (Figure 5D). Altogether, these data showed a significant inhibition of tumor growth associated with a significant reduction in angiogenesis, indicating that the antitumoral effect of 14 kDa hGH could be associated with a reduction in tumoral vasculature, suggesting a potential antiangiogenic effect of 14 kDa hGH in vivo. 

### 2.6. The 14 kDa hGH Inhibits Lung Metastasis In Vivo 

**Figure 6:**
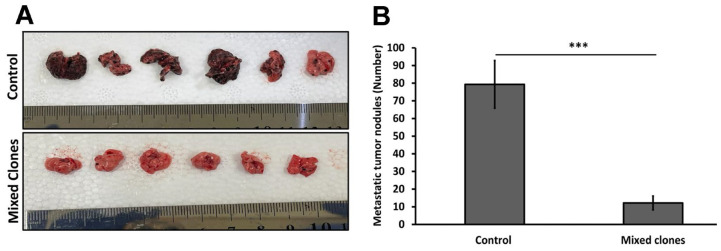
Mice were injected intravenously with either B16-F10 transfected with 14 kDa hGH (mixed clones) or an empty pcDNA3 vector (control), and metastatic lung nodules were quantified 19 days after IV injection. (**A**) Representative pictures of lungs with metastatic nodules harvested from mice injected with 14 kDa hGH-expressing cells (mixed clones) or cells expressing empty pcDNA3 vectors (control). (**B**) The average number of metastatic surface nodules in the lungs from the two groups of mice (n = 6). Data represent mean ± SEM for six mice. Statistically significant differences were calculated at *** *p* < 0.001 versus control.

To further determine the role of 14 kDa hGH on the metastatic outgrowth of tumors, an in vivo lung metastasis assay was performed. B16-F10 cells stably transfected with 14 kDa hGH (mixed clones) or an empty pcDNA3 plasmid (control) were intravenously injected into C57BL/6 mice. Nineteen days postinoculation, mice were sacrificed, their lungs were harvested, and the metastatic nodules on the lung surface were counted. As shown in Figure 6A, there is a notable difference in the number of metastatic tumor nodules (black spots) on the lungs retrieved from control mice (top) compared to the number of nodules covering the lungs of mice injected with 14 kDa hGH-expressing cells (bottom), which showed almost no noticeable nodules, confirmed by a significant decrease in the number of macrometastatic nodules (Figure 6B). Thus, the results clearly verify the antimetastatic effects of 14 kDa hGH in vivo.

### 2.7. The 14 kDa hGH Requires Plasminogen Activator Inhibitor Type-1 to Mediate Its Antiangiogenic Function 

**Figure 7:**
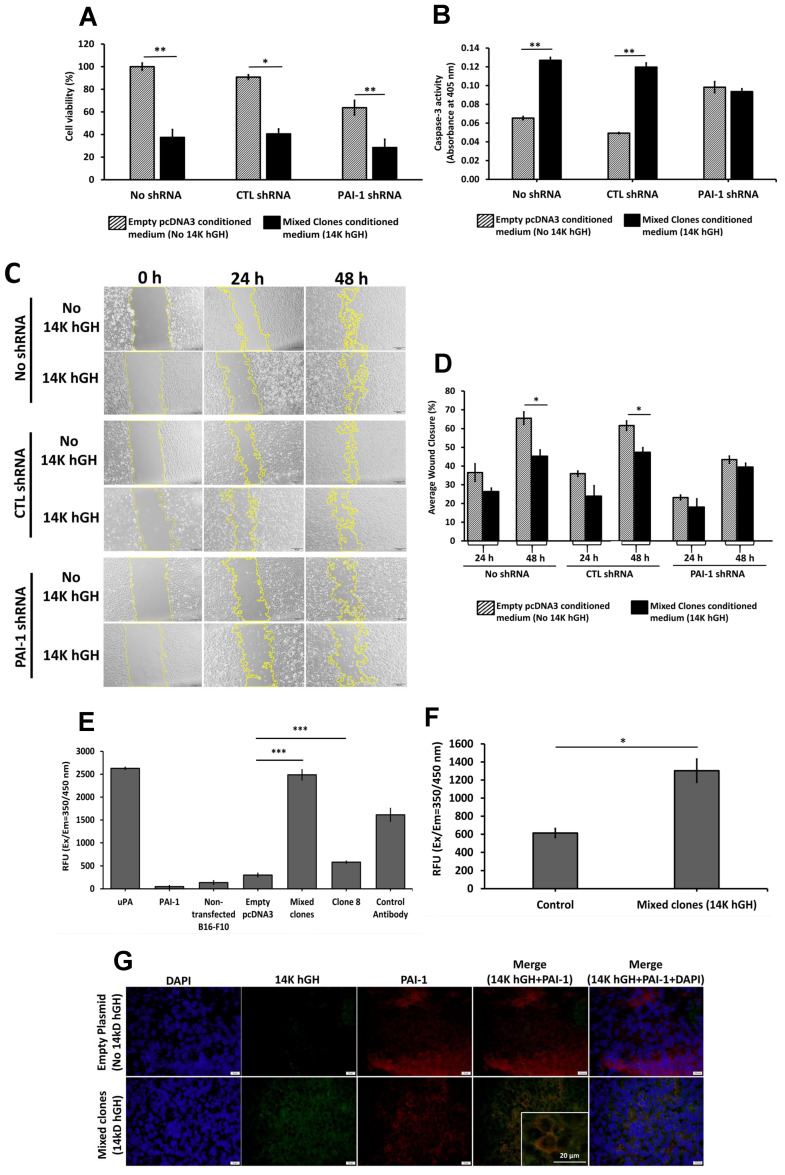
(**A**) Percentage of cell viability using MTT assay, and (**B**) caspase-3 activity of HBME cells transfected with control shRNA or PAI-1 shRNA and then treated with B16-F10 empty pcDNA3 vectors (control) or B16-F10 mixed clones (14 kDa hGH) in conditioned media for 48 h. (**C**) Representative pictures of the scratch assay using HBME cells after transfection with control shRNA or PAI-1 shRNA and treated with B16-F10 empty pcDNA3 vectors (control) or B16-F10 mixed clones (14 kDa hGH) conditioned media. (**D**) The histogram represents the percentage of wound healing after 48 h relative to 0 h. Urokinase activities in (**E**) conditioned media of stably transfected B16-F10 cells with 14 kDa hGH or empty pcDNA3 vectors and (**F**) tumors retrieved from C57BL/6 mice implanted with B16-F10 cells stably transfected with empty pcDNA3 vectors (control, n = 3) or 14 kDa hGH (mixed clones, n = 3) after a 1-h incubation. (**G**) Representative histopathologic images showing immunofluorescence colocalization of 14 kDa hGH (green) with PAI-1 (red) on the cell membrane of tumors retrieved from control mice (**top**) and 14 kDa hGH-bearing mice (**bottom**). Cell nucleus immunostained with DAPI (blue). Scale bars, 20 μm. Data represent the mean ± SD of triplicate wells. Statistically significant differences were calculated at * *p* < 0.05, ** *p* < 0.01, and *** *p* < 0.001 versus empty pcDNA3 control and control shRNA.

Since we previously demonstrated that 16kD prolactin requires PAI-1 to inhibit tumor angiogenesis [12], we investigated the possible involvement of PAI-1 in the antiangiogenic effects of 14 kDa hGH. Stably transfected HBME cells with PAI-1 shRNA and their corresponding controls were used to assess the effect of 14 kDa hGH on endothelial cell proliferation, migration, and apoptosis. Conditioned media from B16-F10 14 kDa hGH (mixed clones) significantly reduced cell proliferation and migration and induced apoptosis in nontransfected cells or cells transfected with control shRNA (Figure 7A–D). However, cells transfected with PAI-1 shRNA and then treated with B16-F10 14 kDa hGH (mixed clones) conditioned media did not display an increase in the levels of apoptotic cells nor a reduction in cell migration (Figure 7B–D), suggesting that the downregulation of PAI-1 expression by PAI-1 shRNA could abolish the proapoptotic and antimigratory effects of 14 kDa hGH. Nonetheless, the downregulation of PAI-1 did not mitigate the antiproliferative effect of 14 kDa hGH (Figure 7A). One of the major functions of PAI-1 is to inhibit uPA and tPA activities; thus, to assess the role of 14 kDa hGH in inhibiting the antiproteolytic activity of PAI-1, B16-F10 14 kDa hGH-conditioned media and tumor extracts were employed. In fact, 14 kDa hGH suspended in B16-F10 14 kDa hGH conditioned media was able to abolish the inhibitory activity of PAI-1 against uPA and show more efficient inhibitory effects than the positive control antibody (Figure 7E). The inhibitory effect of 14 kDa hGH on PAI-1 activity was further confirmed in vivo using tumor protein extracts from B16-F10 cells transfected with 14 kDa hGH, which showed a significant increase in uPA activity compared to control tumor extracts (Figure 7F). To further investigate the in vivo interaction between 14 kDa hGH and endogenous mouse PAI-1, immunofluorescence staining was performed to evaluate 14 kDa hGH and PAI-1 colocalization in tumors retrieved from C57BL/6 mice implanted with B16-F10 cells stably transfected with 14 kDa hGH (mixed clones) compared to tumors retrieved from C57BL/6 mice implanted with B16-F10 cells stably transfected with empty pcDNA3 vectors (control). The 14 kDa hGH colocalizes with PAI-1 on the tumor cell membrane in 14 kDa hGH mixed clones. However, the control tumor showed no 14KhGH staining, and only endogenous PAI-1 was positively stained (Figure 7G). 

## 3. Discussion

The 14 kDa hGH fragment belongs to the family of vasoinhibins, in which the members of this family share similar structural and functional properties and exert various effects on endothelial cells, such as inhibiting vasodilation, vasopermeability, and angiogenesis [20,21]; therefore, we are interested in investigating the role of the 14 kDa human growth hormone that has not been extensively studied compared to the 16kDa fragment of prolactin [11,12,22,23,24,25,26,27], and to understand their underlying mechanisms of action.

In the present study, we demonstrated the antitumoral, antiangiogenic, and antimetastatic functions of 14 kDa hGH using complementary in vitro and in vivo models. In vitro, 14 kDa hGH instigated an inhibition of cellular proliferation, migration, and angiogenesis and induced cellular apoptosis in B16-F10 tumor cells. Furthermore, we confirmed the antiangiogenic functions of 14 kDa hGH on HBME cells, as indicated by a significant decrease in cell survival, cell migration, tube formation abilities, and a notable increase in cellular apoptosis. In concordance with previous studies demonstrating the role of 16kD PRL in reducing B16-F10, HCT116, and PC-3 tumor cell growth in vivo [11,28,29], we have shown that 14 kDa hGH significantly reduces primary tumor growth and metastasis in C57BL/6 mice. The diminution in tumor growth was accompanied by a marked reduction in the number of tumor blood vessels and a change in vessel morphology determined by vessel immunostaining and quantification on tumor sections, suggesting that decreased blood vessel formation may contribute to reduced tumor growth after 14 kDa hGH release from the B16-F10 14 kDa hGH tumors. Similarly, the lungs of mice subcutaneously injected with B16-F10 14 kDa hGH showed almost no tumor nodules compared to their corresponding controls. This reduction in the number of tumor nodules could be attributed either to a direct effect of 14 kDa hGH on tumor cells or an indirect reduction in angiogenesis.

Since we previously demonstrated a major role of PAI-1 in mediating the antiangiogenic effects of 16kDa PRL [12], we treated HBME cells stably transfected with PAI-1 shRNA with conditioned media collected from B16-F10 14 kDa hGH transfected cells. The downregulation of PAI-1 expression in HBME cells abolished the antimigratory and proapoptotic effects of 14 kDa hGH and confirmed the key role of PAI-1 in mediating the antiangiogenic function of 14 kDa hGH. However, the reduction in PAI-1 expression did not affect the 14 kDa hGH antisurvival activity.

Further validation of the PAI-1/14 kDa hGH interaction hypothesis is the inhibitory effect of 14 kDa hGH-rich conditioned media on recombinant PAI-1 activity and the increase in uPA activity in 14 kDa hGH tumors compared to control tumors. Those data corroborate the previously described function of PAI-1 as a major fibrin scaffold stabilizer for endothelial cell growth and motility [30]. The stable downregulation of PAI-1 expression in HBME cells was associated with a significant reduction in cellular proliferation and migration and an increase in cellular apoptosis, which confirms our previous study [31].

Several antiangiogenic molecules were developed, such as anti-VEFG, with high potential for therapeutic use but with substantial side effects [32]. The 14 kDa hGH is generated by proteolytic cleavage of the growth hormone. The same applies to angiostatin and endostatin, which are derived from plasminogen and collagen cleavage, respectively. Those molecules are strong antiangiogenic molecules with fewer side effects [33]. Therefore, targeting cancer using 14 kDa hGH or its derived peptides will have fewer side effects since few physiological functions are associated with 14 kDa hGH. Subsequently, 14 kDa hGH will require more extensive studies to establish its efficiency and safety compared to other antitumoral and antiangiogenic molecules. 

Contrary to the full-length growth hormone with various vital physiological functions in the organism, the generated 14 kDa hGH does not induce the same effects as its full-length counterpart, and its physiological effects are limited to vasopermeability, vasodilation, and angiogenesis. In our experiment, 14 kDa hGH was expressed by tumor cells to ensure local expression of the molecule. However, a systemic expression of 14 kDa GH would be possible using an adenovirus vector, as we previously performed using 16kDa PRL [11]. Since we showed that 14 kDa hGH requires PAI-1 to mediate its antitumoral and antiangiogenic effects, the levels of PAI-1 expressed by tumor cells or in the tumor microenvironment will be a determinant factor for the efficiency of 14 kDa hGH. Therefore, testing 14 kDa hGH in additional tumor models will validate our finding.

It is well established that high levels of PAI-1 are correlated with poor prognosis in several types of tumors [34], which could be explained by its proangiogenic functions [12,35]. Inhibiting PAI-1 with 14 kDa hGH could be one of the mechanisms by which 14 kDa hGH could mediate its antiangiogenic effects. We previously demonstrated that 16kDa PRL requires PAI-1 to induce its antiangiogenic and antitumoral effects and that PAI-1 is a binding partner for 16kDa PRL responsible for the internalization of the 16kD PRL into endothelial cells [12]. Our data showed the colocalization of 14 kDa hGH and PAI-1 on the cell membrane of tumor cells expressing 14 kDa hGH. The colocalization in the tumor tissues could be preliminary evidence of a direct interaction between the two molecules. However, the affinity and dynamic interaction between 14 kDa GH and PAI-1 require additional experiments, and the mechanism regarding 14 kDa hGH internalization remains unclear and requires further investigation.

In conclusion, we demonstrated for the first time that 14 kDa hGH attains antitumoral, antimetastatic, and antiangiogenic functions and could be used as a new therapeutic molecule. We also provide preliminary evidence for the involvement of PAI-1 in its antiangiogenic functions. Further studies are needed to deeply understand its mechanism of interaction with PAI-1 in the pathology of cancer and other pathologies related to high levels of PAI-1. 

## 4. Materials and Methods

### 4.1. Plasmid Construction

The 14 kDa hGH cDNA sequence was digested from the expression vector Pshuttle2-hGH14K by KpnI and NdeI restriction enzymes (Promega, Madison, WI, USA) and subcloned into KpnI and NdeI restriction sites in the pcDNA3 vector. The newly constructed expression vector contains an ampicillin and neomycin resistance gene cassette, allowing for the selection and maintenance of the transfected clones. Subsequently, the pcDNA3 expression vector was expanded using DHa5-competent E. coli and extracted using the maxiprep plasmid extraction kit (Sigma-Aldrich, MO, USA) according to the manufacturer’s protocol. Sanger sequencing was performed using the BigDye Terminator v3.1 Cycle Sequencing Kit (Applied Biosystems, Thermo Fisher Scientific, Waltham, MA, USA) according to the manufacturer’s protocol to verify the incorporation of 14 kDa hGH insert in the correct orientation within the pcDNA3 plasmid vector. An empty pcDNA3 plasmid vector was used as a negative control.

### 4.2. Cell Culture

B16-F10 mouse melanoma cells were acquired from the American Type Culture Collection (CRL-6475, Rockville, MD) and cultured in Dulbecco’s modified eagle medium-high glucose containing 10% fetal bovine serum (FBS) and 1% penicillin/streptomycin (all from Sigma-Aldrich, saint louis, MO, USA). Human brain microvascular endothelial cells (HBMECs) were surgically isolated from resected brain tissues of children with seizure disorders, as previously described [36]. The cells were maintained in RPMI-1640 medium with 10% fetal bovine serum (FBS) and 1% penicillin-streptomycin (Pen/Strep) (all from Sigma-Aldrich, saint louis, MO, USA). Both cell lines were incubated in a 5% CO_2_ humidified chamber at 37 °C.

### 4.3. Cell Transfection

B16-F10 and HBME cells were seeded at a density of 8 × 10^5^ cells/60 mm^2^ culture plate. After reaching 80% confluency, 11 µg of the control pcDNA3 or the pcDNA3-14 kDa hGH was used to transfect B16-F10 cells or HBME cells using Lipofectamine™ 3000 according to the manufacturer’s instructions (Invitrogen, Carlsbad, CA, USA). Stably transfected B16-F10 cells were then selected using 800 µg/mL of geneticin (G418; InvivoGen, Carlsbad, CA, USA). Individual clones were picked out, grown to confluence, and expanded for further analyses; the remaining resistant cells were allowed to grow and amalgamate to form the mixed clones. The HBME cells were used 48 h after transient transfection with the control pcDNA3 plasmid or the pcDNA3-14 kDa hGH plasmid. Short-hairpin RNA was used to suppress the secretion of PAI-1 in HBME cells using the pSIH-H1 cloning and expression Lentivectors Kit (System Biosciences, Palo Alto, CA, USA) following the manufacturer’s protocol. Human PAI-1 cDNA (GenBank accession number X12701) (5′-AAGGACGAGATCAGCACCACA-3′) or scrambled sequence as previously described [31] were used to transfect HBME cells with Lipofectamine™ 3000 according to the manufacturer’s protocols (Invitrogen, Carlsbad, CA, USA). Copepod green fluorescent protein (copGFP) was used as a reporter for the transfected/transduced cells. The cells with higher shRNA expression levels were selected after two copGFP cell sortings.

### 4.4. Reverse Transcription and Real-Time PCR

Total RNA was extracted from B16-F10 and HBME cells using RNeasy Mini Kit (Qiagen, Hilden, Germany) according to the manufacturer’s protocols. Then, 500 ng of total RNA were used to synthesize cDNA using SuperScript™ First-Strand Synthesis System (Invitrogen, Carlsbad, CA, USA) following the manufacturer’s instructions. Next, cDNA was used for reverse transcription or qPCR using REDTaq ^®^ ReadyMix™ PCR Reaction Mix (Sigma-Aldrich, Saint Louis, MO, USA) or 5x HOT FIREPol^®^ EvaGreen^®^ qPCR Supermix (Solis BioDyne, Tartu, Estonia), respectively. The following primers were used: mouse β-actin (Internal control), forward: 5’-AGCTTCTTTGCAGCTCCTTC-3’, reverse: 5’-CCACCATCACACCCTGGT-3’; 14 kDa hGH, forward: 5’-CAGTGCCTTCCCAACCATTC-3’, reverse: 5’-GGAGCAGCTCTAGGTTGGAT-3’. The relative expression of the 14 kDa hGH gene was calculated according to the $2^{-\Delta \Delta C\left(t\right)}$ method. The reverse transcription and qPCR were performed in three separate wells for each condition.

### 4.5. Protein Extraction and Western Blotting

Transfected B16-F10 and HBME cells were harvested and lysed using an ice-cold lysis buffer containing 150 mM NaCl, 50 mM Tris-HCl, 5 mM EDTA, 1% Triton X-100, 10% glycerol, and 1x Protease and Phosphatase Inhibitor Cocktail (Thermo Fisher Scientific, Waltham, MA, USA). Cell lysates and B16-F10 conditioned media were centrifuged at 18,000× *g* for 15 min at 4 °C. Soluble protein concentrations in cell lysates were then quantified using Micro BCA™ Protein Assay Kit (Thermo Fisher Scientific) following the manufacturer’s protocols. Denatured protein samples were resolved on 12% SDS-PAGE gel and then transferred onto a nitrocellulose membrane (Bio-Rad, Hercules, CA, USA). Equal protein loading was subsequently confirmed using Ponceau S staining solution. Thereafter, the membranes were blocked overnight at 4 °C with 5% skimmed milk in Tris-Buffered Saline Buffer with Tween^®^ 20 (TBST), followed by incubation for 2 h with primary antibodies: rabbit polyclonal anti-14 kDa hGH antibody (1:1000 dilution; in-house developed; a gift from Dr. Jean Closset, University of Liege, Belgium) and rabbit monoclonal anti-β-actin antibody (1:1000 dilution; 4970; Cell Signaling Technologies, Danvers, MA, USA). Subsequently, the membranes were incubated with HRP-conjugated goat antirabbit IgG secondary antibody (1:1000 dilution; 7074; Cell Signaling Technologies, Danvers, MA, USA) for 1 h at room temperature. The immunoblots were visualized on ChemiDoc Imaging Systems (Bio-Rad, Hercules, CA, USA) using Clarity™ Western ECL Substrate (Bio-Rad, Hercules, CA, USA).

### 4.6. Cell Viability Assay

Cell proliferation was quantified using the MTT assay [bromide-soluble tetrazolium dye, 3-(4,5-dimethylthiazol-2-yl)-2,5-diphenyl tetrazolium] (Sigma-Aldrich, MO, USA). Transfected B16-F10 and HBME cells with the control pcDNA3 plasmid or the pcDNA3-14 kDa hGH plasmid (3 × 10^3^ cells/well) were seeded in triplicates into a 96-well plate. In addition, HBME cells transfected with control plasmid or PAI-1 shRNA plasmid were seeded at a density of 5 × 10^3^ cells/well and treated with B16-F10 empty pcDNA3 (control) or B16-F10 mixed clones (14 kDa hGH) conditioned media for 48 h. After 48 h of incubation, the MTT reagent (5 mg/mL) was loaded onto the cells and incubated for 3 h in a humid chamber with 5% CO_2_ at 37 °C. After 3 h, the produced formazan crystals were solubilized using dimethyl sulfoxide (DMSO). The color absorbance was measured at 570 nm wavelength using a microplate reader. Cell viability was normalized to the nontransfected cells used as controls.

### 4.7. Cell Apoptosis

The degree of apoptosis in B16-F10 and HBME cells was evaluated using the Annexin V-FITC Apoptosis Detection Kit (Thermo Fisher Scientific, MA, USA) according to the manufacturer’s protocol. Briefly, floating and attached cells were collected 48 h postincubation, washed with PBS, suspended in Annexin-V binding buffer, and stained with Propidium Iodide and Annexin V-FITC. Stained cells were then analyzed using the BD FACSAria III cell sorter at 475/650 nm excitation/emission spectra (BD Biosciences, Franklin Lakes, NJ, USA). The results were analyzed using FlowJo software (BD Bioscience, NJ, USA). The experiment was performed in triplicates for each condition and compared to cells transfected with empty pcDNA3 plasmid using a 2-tailed student’s t-test.

### 4.8. Soft Agar Colony Formation Assay

Anchorage-independent soft agar colony formation assay was implemented as previously described [37]. Briefly, B16-F10 cells (5 × 10^3^) were mixed with a complete growth medium and 0.6% noble agar and then placed on top of a 1% noble agar layer in each well of a 6-well plate. The plate was incubated at room temperature in a culture hood for 30 min, allowing the cell/agar mixture to solidify. Cells were maintained for 21 days in a humidified chamber at 37 °C, with fresh medium replacement twice weekly. Colonies were stained with 0.5% crystal violet in 25% methanol, photographed, and counted in triplicate wells. Cells transfected with empty pcDNA3 plasmid were considered as a control condition.

### 4.9. Cell Migration

Stably transfected B16-F10 clones, transiently transfected HBME cells, and stably transfected HBME cells with control plasmid or PAI-1 shRNA plasmid were then treated with B16-F10 empty pcDNA3 (control) or B16-F10 mixed clones (14 kDa hGH) conditioned media for 48 h and cultured in 6-well plates at 2.5 × 10^5^ cells/well seeding density. At 90% confluency, a linear wound was created by vertically scratching the confluent cell monolayer using a sterile 200 µL micropipette tip. Images were then captured at fixed positions along the wound at 0, 24, and 48 h using Olympus cellSens imaging software (Olympus, Tokyo, Japan). Pictures were successively analyzed for percent wound closure using ImageJ software. The average value for each condition is the mean of three measurements taken from three separate wells. 

### 4.10. Matrigel Angiogenic Assay 

Matrigel (BD Biosciences, NJ, USA) was added at 50 µL/well of a 96-well plate and incubated for 30 min in a humidified incubator at 37 °C allowing the Matrigel to polymerize. B16-F10 and HBME cells (2 × 10⁴) were suspended with appropriate growth media and loaded on top of the Matrigel-coated wells in triplicates for each condition. Cells were incubated overnight at 37 °C, and pictures were acquired from five independent fields per well using Olympus cellSens imaging software (Olympus, Tokyo, Japan) to quantify the number of tubes in triplicate wells. The average value for each condition is the mean of three tube numbers taken from three separate wells. Cells transfected with empty pcDNA3 plasmid were considered as a control condition.

### 4.11. Mice 

Female C57BL/6 mice (six- to eight-week-old) were obtained and maintained in the Animal Facility of the Sharjah Institute for Medical Research at the University of Sharjah (SIMR, Sharjah, UAE). All experimental mouse procedures were performed in accordance with the standard Public Health Service and international standard protocols and approved by the Animal Care and Use Committee of the University of Sharjah, Sharjah, UAE.

### 4.12. In Vivo Tumorigenicity 

The B16-F10 syngeneic tumor model was used for in vivo tumorigenicity assay. Groups of 14 mice were randomly divided and allocated into the control group (cells transfected with empty pcDNA3 plasmid, n = 7) and the mixed clone groups (cells transfected with a pcDNA3-14 kDa hGH plasmid, n = 7). For the experimental procedure, mice were anesthetized with isoflurane (Wellona Pharma, Gujarat, India) by inhalation. B16-F10 cells (1 × 10^6^ cells in 100 µL PBS) were injected subcutaneously into the right dorsal flank of mice using a 1 mL syringe with a 30-gauge needle. Tumor sizes (length and width) were measured with a vernier caliper every seven days after tumors became palpable, and tumor volume was calculated using the following formula: length × width^2^ × 0.5. Mice had free access to food and water and were monitored for signs of illness or infection. Mice were sacrificed via cervical dislocation 21 days postinoculation, and their tumors were surgically resected and weighed. Tumor specimens were cut into two halves; one half was frozen in cryomolds immersed in polyfreeze tissue freezing medium (Sigma-Aldrich, MO, USA) and stored at −80 °C for tissue sectioning and immunohistological analyses, and the other half was used for protein extraction using the Dounce Homogenizer for further analyses.

### 4.13. Immunohistological Analysis of Tumor Vascularization and Colocalization of 14 kDa hGH and PAI-1

Seven tumors were retrieved from C57BL/6 mice from each group: control (empty pcDNA3) and mixed clones (14 kDa hGH) to prepare the tumor tissue sections, perform immunostaining, and determine the average number of vessels. Tumor vascularity, angiogenesis, and immunofluorescence colocalization were assessed on the frozen tumor tissue sections. Tumor vascularity, angiogenesis, and immunofluorescence colocalization were assessed on frozen tumor tissue sections. Cryosections (6-µm thick) were rehydrated and blocked with 5% bovine serum albumin (BSA), followed by incubation with primary antibodies: rabbit polyclonal anticollagen IV antibody (1:100 dilution; in-house developed; University of Liege, Belgium), rabbit polyclonal anti-14 kDa hGH antibody (1:100 dilution; in-house developed; a gift from Dr. Jean Closset, University of Liege, Belgium), and mouse monoclonal anti-PAI-1 antibody (1:100 dilution; MA-33H1F7; in-house developed; a gift from Professor Paul Declerck, University of KU Leuven, Belgium) for 1 h at room temperature. After three washes with TBS for 10 min, the slides were incubated with secondary antibodies: Cy3 goat antirabbit antibody (1:100 dilution; 111-165-144 for collagen IV; Jackson ImmunoResearch, West Grove, PA, USA), FITC donkey antirabbit antibody (1:100 dilution; for 14 kDa hGH; Vector Laboratories, CA, USA), Texas Red Horse antimouse IgG antibody (1:100 dilution; TI20001.5 for PAI-1; Vector Laboratories, CA, USA), and DAPI for 1 h at room temperature. Following incubation, the slides were washed, mounted with Fluoroshield™ Histology Mounting Medium (Sigma-Aldrich, MO, USA), and viewed under a fluorescence microscope. Eight to ten fields were examined per section, and the number of vessels/mm^2^ was quantified using Olympus cellSens imaging software (Olympus, Tokyo, Japan). A 2-tailed student’s t-test was used to determine the statistical difference between the two groups.

### 4.14. In Vivo Pulmonary Metastasis Model

The B16-F10 syngeneic tumor model was used for the in vivo pulmonary metastasis assay. Additionally, 5 × 10^5^ cells in 100 µL PBS of B16-F10 control cells (transfected with empty pcDNA3 plasmid) and B16-F10 mixed clone cells (transfected with the pcDNA3-14 kDa hGH plasmid) were inoculated intravenously into the lateral tail vein of C57BL/6 mice (n = 6/group). Mice had free access to food and water and were monitored for signs of illness or infection. Nineteen days after inoculation, mice were sacrificed, lungs were harvested, and the average number of metastatic colonies on the lung surface of each group (n = 6) was counted and reported as a mean value. A 2-tailed student’s t-test was used to determine the statistical difference between the two groups.

### 4.15. Urokinase Activity Assay

Conditioned media from stably transfected B16-F10 with empty pcDNA3 or pcDNA3-14 kDa hGH were collected, lyophilized, and reconstituted in proportional quantities of PBS. In addition, tumors retrieved from C57BL/6 mice implanted with B16-F10 cells stably transfected with empty pcDNA3 vector (control, n = 3) or 14 kDa hGH (mixed clones, n = 3) were homogenized using Dounce Homogenizer, and total proteins were extracted using RIPA lysis buffer (Sigma-Aldrich, MO, USA) and quantified using Micro BCA™ Protein Assay Kit (Thermo Fisher Scientific, MA, USA) following the manufacturer’s protocols. The urokinase activity assay kit (Abcam, Cambridge, UK) was used to assess the amount of urokinase-type plasminogen activator (uPA) in 48 µL of the conditioned media, and 48 µL of tissue lysates at 1 mg/mL following the manufacturer’s protocols. In a 96-well white plate, triplicates of each condition medium were incubated or not with 2.2 µg/mL uPA, 20 µg/mL PAI-1, and 300 µg/mL anti-PAI-1, and the fluorescence was measured at Ex/Em = 350/450 nm using GloMax Discover microplate reader (Promega, Madison, WI, USA) after 1 h of incubation at room temperature.

### 4.16. Caspase-3 Activity Assay

The activity of caspase-3 was measured using the Caspase-3 Colorimetric Assay (Abcam, Cambridge, UK) according to the manufacturer’s instructions. Briefly, HBME cells transfected with control plasmid or PAI-1 shRNA plasmid were cultured in a 60-mm culture dish at 8 × 10^5^ cells seed density, then treated with B16-F10 empty pcDNA3 (control) or B16-F10 mixed clones (14 kDa hGH) conditioned media for 48 h. After 48 h of treatment, cells were collected and lysed in 50 µL of the supplied lysis buffer. Then, 100 µg of cell lysate proteins were suspended in 50 µL of reaction buffer containing DEVD-p-NA substrate and 10 mM dithiothreitol (DTT) and incubated at 37 °C for 2 h. The reactions were measured at 405 nm using the GloMax Discover microplate reader (Promega, Madison, WI, USA).

### 4.17. Statistical Analysis

In vitro experiments were performed in triplicates of an independent assay and repeated at least two to three times; data were represented as mean ± standard deviation (SD). For in vivo experiments, data were represented as means ± standard error (SEM). Analyses for statistical significance were determined using a 2-tailed student’s *t*-test. *p* values < 0.05 were considered significant.

## Data Availability

The authors declare that all data generated or analyzed during this study are available within the published article.
